# Longitudinal assessment of lung clearance index to monitor disease progression in children and adults with cystic fibrosis

**DOI:** 10.1136/thoraxjnl-2021-216928

**Published:** 2021-07-22

**Authors:** Alex R Horsley, John Belcher, Katie Bayfield, Brooke Bianco, Steve Cunningham, Catherine Fullwood, Andrew Jones, Anna Shawcross, Jaclyn A Smith, Anirban Maitra, Francis J Gilchrist

**Affiliations:** 1 Division of Infection, Immunity and Respiratory Medicine, The University of Manchester Faculty of Biology, Medicine and Health, Manchester, UK; 2 Manchester Adult Cystic Fibrosis Centre, Manchester University NHS Foundation Trust, Manchester, UK; 3 School of Medicine, Keele University, Keele, UK; 4 Respiratory Medicine, Children's Hospital at Westmead, Westmead, New South Wales, Australia; 5 MRC Centre for Inflammation Research, University of Edinburgh, Edinburgh, UK; 6 Statistics, Research and Innovation, Manchester University NHS Foundation Trust, Manchester, UK; 7 MAHSC Centre for Biostatistics, University of Manchester, Manchester, UK; 8 Royal Manchester Children's Hospital, Manchester University NHS Foundation Trust, Manchester, UK; 9 Academic Department of Child Health, University Hospitals of North Midlands NHS Trust, Stoke-on-Trent, UK; 10 Institute of Applied Clinical Sciences, Keele University, Keele, UK

**Keywords:** cystic fibrosis, lung physiology

## Abstract

**Background:**

Lung clearance index (LCI) is a valuable research tool in cystic fibrosis (CF) but clinical application has been limited by technical challenges and uncertainty about how to interpret longitudinal change. In order to help inform clinical practice, this study aimed to assess feasibility, repeatability and longitudinal LCI change in children and adults with CF with predominantly mild baseline disease.

**Methods:**

Prospective, 3-year, multicentre, observational study of repeated LCI measurement at time of clinical review in patients with CF >5 years, delivered using a rapid wash-in system.

**Results:**

112 patients completed at least one LCI assessment and 98 (90%) were still under follow-up at study end. The median (IQR) age was 14.7 (8.6–22.2) years and the mean (SD) FEV_1_ z-score was −1.2 (1.3). Of 81 subjects with normal FEV_1_ (>−2 z-scores), 63% had raised LCI (indicating worse lung function). For repeat stable measurements within 6 months, the mean (limits of agreement) change in LCI was 0.9% (−18.8% to 20.7%). A latent class growth model analysis identified four discrete clusters with high accuracy, differentiated by baseline LCI and FEV_1_. Baseline LCI was the strongest factor associated with longitudinal change. The median total test time was under 19 min.

**Conclusions:**

Most patients with CF with well-preserved lung function show stable LCI over time. Cluster behaviours can be identified and baseline LCI is a risk factor for future progression. These results support the use of LCI in clinical practice in identifying patients at risk of lung function decline.

**Key messages**

**What is the key question?**

- In patients with mild cystic fibrosis (CF) lung disease, how does lung clearance index (LCI) change over time and what risk factors are associated with this?

**What is the bottom line?**

- In most patients with predominantly mild CF, LCI remains stable over time.
- Worsening LCI was associated with higher baseline LCI, increased age, lower baseline FEV_1_, *Pseudomonas* acquisition and increased intravenous antibiotic courses.

**Why read on?**

- LCI can be a powerful and sensitive tool in CF and may be especially important in the postmodulator era, but has been hard to implement clinically.
- This study is the first to address long-term LCI trajectory in those with predominantly mild disease and also addresses some of the practical issues around routine LCI testing.

## Introduction

Lung clearance index (LCI) derived from the multiple breath washout (MBW) test is an established research outcome for individuals with cystic fibrosis (CF).^1–3^ The test involves following an inert gas washed out from the lungs during tidal breathing. This makes it simple to perform from a patient’s perspective and correspondingly applicable to a wide range of ages and disease states.^4 5^ Key advantages of LCI over FEV_1_ include increased sensitivity to early changes in airway obstruction,^6^ ability to be performed repeatedly even in very young children^7 8^ and a very stable upper limit of normal, even in growing lungs.^9 10^ LCI is recognised as a valuable endpoint in CF clinical trials, with guidelines for the performance and interpretation of the test,^2 5 11^ and has played an important role in supporting registration of novel cystic fibrosis transmembrane conductance regulator (CFTR) modulator therapies.^3 12 13^

Despite this increased acceptance and use of LCI in research, the technique has yet to become established in routine clinical care. From a technical perspective, the test takes more time than spirometry to perform (to which it would be additional) and the equipment is poorly mobile, making the test difficult to integrate into multidisciplinary CF clinic scheduling.^14^ Key clinical questions remain relating to natural variability in LCI, progression over time and what constitutes a minimally important clinical change in LCI within patients.

To address these challenges and enable integration of LCI into clinical practice, we performed "LCI-SEARCH", a National Institute for Health Research-funded multicentre, all-age, prospective study of the value of LCI in clinical CF practice.^15^ To reduce technical barriers, we employed a closed-circuit system which delivers more rapid wash-in, reducing overall test time, as well as allowing the system to be fully mobile.^10^ Since LCI appears to have most value in those with mild disease, this study specifically focused on routine follow-up in children and adults with well-preserved FEV_1_ who were considered free of *Pseudomonas*. The objectives of this study were the following:

- To evaluate feasibility, acceptability and clinical value of repeated LCI measurements in clinical monitoring in CF outpatient clinics.
- To assess short-term reproducibility in patients with clinically stable CF with predominantly mild disease.
- To assess long-term trajectory of LCI in CF and identify risk factors for accelerated decline.

## Methods

This was a prospective, single-blind, observational study of children (≥5 years) and adults with CF, where routine LCI testing was integrated into clinical care in parallel with conventional clinic-based spirometry. Patients were recruited from three specialist CF centres in the UK: Wythenshawe Hospital, Manchester, Royal Manchester Children’s Hospital and University Hospital North Midlands. Patients had FEV_1_ >50% and were recruited from non-*Pseudomonas* clinics at each site (defined as currently free of chronic infection with *Pseudomonas aeruginosa*).^16^ Those who were subsequently reclassified as chronically infected or displayed new *Pseudomonas* infections during follow-up remained in the study. Patients and parents provided written informed consent and children assent.

### Study visits

Patients were assessed at their usual clinic appointments, including both routine and emergency visits. Patients or parents completed a short questionnaire, comparing current symptoms with usual baseline and identifying any other symptoms of a pulmonary exacerbation.^17^ Study visits took place between November 2014 and February 2018. In order to establish repeatability and individual patient trajectories, LCI data were kept blind until the final 6 months of the study. Following this, they were revealed to clinical teams in real time at each clinic visit, along with a patient-specific report and graph showing all historical LCI data. During this period, clinicians were asked to rate the impact of having these LCI data on clinical decision-making using a 3-point scale (no impact, partial impact, strong impact). Adult patients were provided with a questionnaire to rate their experience of repeated LCI measurement. Details are provided in the online supplemental file.

10.1136/thoraxjnl-2021-216928.supp1 Supplementary data

### Multiple breath washout

MBW was performed using a closed-circuit Innocor system (PulmoTrace ApS, Glamsbjerg, Denmark), as previously described and detailed in the online supplemental file.^10 18^ Detailed analysis and quality control were performed in a separate offline washout analysis package prepared in Igor Pro V.6 (Wavemetrics, Lake Oswego, Oregon, USA), as previously described.^10 12 19 20^ Washout repeats were excluded if there was evidence of leak or a large difference between LCI or functional residual capacity (FRC) measurements (>25% from median).^5^ Final LCI and FRC values are the average of at least two reproducible repeats. The upper limit of normal for LCI was 6.9.^10^

### Statistical analysis

To assess between-test short-term repeatability of LCI, only paired measurements taken within 6 months of each other were included. Patients were required to be clinically stable at both assessments, defined as no additional oral or intravenous antibiotics within 14 days, deemed well by the reviewing physician and FEV_1_ change <10% of previous measurement. Bland-Altman analysis^21^ and intraclass correlation (ICC) coefficient were used to describe change in LCI between visits.

Longitudinal analysis was only performed on those with at least four valid measurements while clinically stable. Latent class growth analysis (LCGA) was used to identify distinct trajectories of LCI data.^22^ This method is described in more detail in online supplemental file. LCGA was undertaken using the LCMM package in R.^23^

Linear mixed modelling with multivariable adjustment was used to investigate clinical factors associated with change in LCI over time. A random effects model with an exchangeable correlation structure was used to estimate the ICC values and limits of agreement. Covariates were chosen a priori to include the following clinical data previously recognised to be associated with lung function decline: *Pseudomonas* status at study start (chronic and intermittent infection vs not infected), intravenous antibiotic courses, all antibiotic-treated exacerbations, age, body mass index and gender. Pancreatic status was added subsequently during data analysis. Baseline LCI and FEV_1_ were included, but in order to reduce the number of factors only FEV_1_ z-score was included of the spirometric indices.

Original sample size was planned to be ≥70 participants. This was based on an estimate of what was required for reasonably robust longitudinal modelling and not calculated against a specific outcome (since no longitudinal data sets existed at the time).

## Results

**Figure 1:**
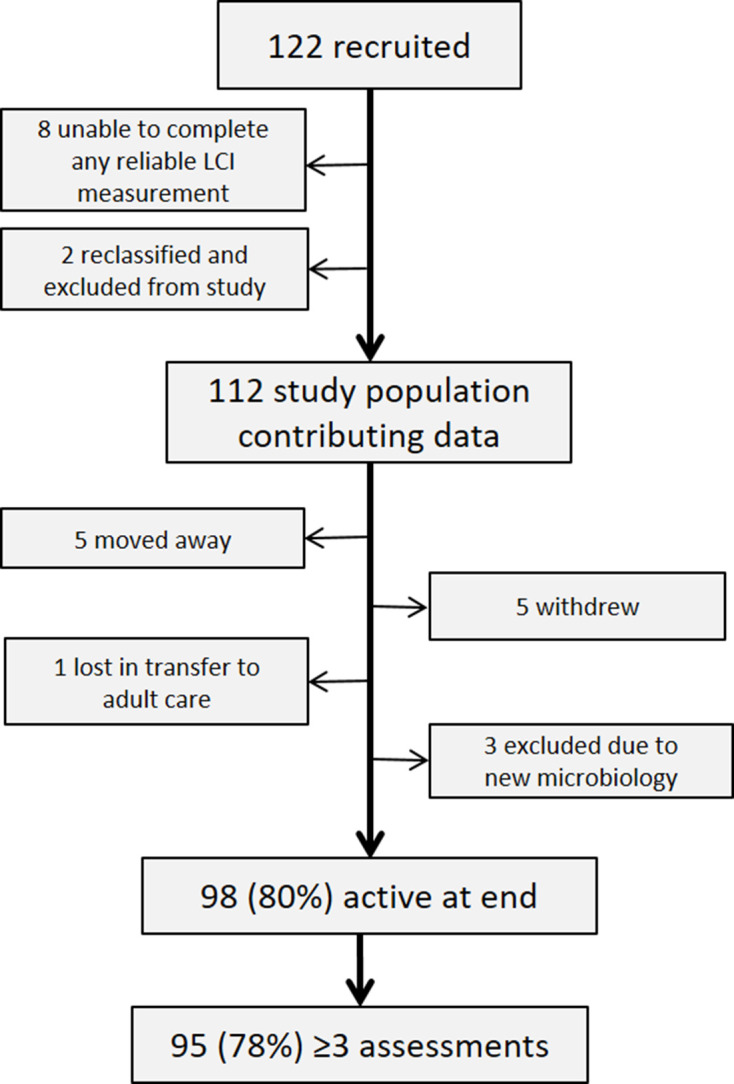
CONSORT diagram showing outcomes from 122 children and adults with cystic fibrosis recruited for longitudinal lung clearance index (LCI) measurements. CONSORT, Consolidated Standards of Reporting Trials.

The study recruited 122 children and adults with CF, of whom 112 (92%) completed at least one successful LCI assessment and 98 (90%) were still under follow-up when the study finished. A CONSORT (Consolidated Standards of Reporting Trials) diagram of patient outcomes is shown in figure 1 and in the online supplemental file.

Summary demographic and clinical data for those with at least one LCI measurement are shown in table 1. The median (range) age at study entry was 14.7 (5.1–65.3) years. Forty-four subjects were adults (>18 years) assessed at the adult CF centre and 68 were children. Two subjects transitioned to adult care and had measurements performed at both paediatric and adult centres.

**Table 1:** Baseline demographic and clinical characteristics of patients with cystic fibrosis with at least one successful LCI measurement

	All subjects	Children (<18 years)	Adults	P value^*^
n	112	68	44	
Male:female	64:48	38:30	26:18	0.85
Age (IQR), years	14.7 (8.6–22.2)	9.2 (7.2–12.1)	24.5 (21.6–29.3)	
Mean FEV_1_ % (SD)	85.3 (16.0) (n=110)	87.6 (14.4)	81.7 (18.8)	0.054
Mean FEV_1_ z-score (SD)	−1.2 (1.3)	−1.0 (1.1)	−1.5 (1.4)	**0.044**
Mean FVC z-score	−0.6 (1.1)	−0.5 (1.0)	−0.8 (1.2)	0.107
Mean FEF_25–75_ z-score	−1.2 (1.5)	−0.9 (1.5)	−1.6 (1.4)	**0.018**
Median BMI (IQR), kg/m^2^		z-score: 0.30 (−0.52 to 1.05)	23.2 (21.2–26.5)	n/a
Phe508del homozygotes, n (%)	50 (45)	39 (57)	11 (25)	**0.0009**
Median sweat chloride, mmol/L (IQR)	99 (85–108) (n=66)	100 (86–110) (n=51)	87 (71–98) (n=15)	0.056
Median age at diagnosis (IQR)	1.0 (0.1–5.3)	0.2 (0–1.5)	5.0 (1.5–16.5)	**<0.0001**
Pancreatic sufficient, n (%)	40 (36)	13 (19)	27 (61)	**0.0001**
Never *Pseudomonas aeruginosa*, n (%)	45 (40)	24 (35)	21 (48)	0.237
Previous *Pseudomonas aeruginosa*, n (%)	17 (15)	13 (19)	4 (9)	0.184
Intermittent *Pseudomonas aeruginosa*, n (%)	47 (42)	30 (44)	17 (39)	0.695
Chronic *Pseudomonas aeruginosa*, n (%)	3 (3)	1 (2)	2 (5)	0.560
Intermittent and chronic *Staphylococcus aureus*, n (%)	57 (51)	31 (46)	26 (59)	0.180
Intermittent and chronic *Aspergillus*, n (%)	19 (17)	11 (16)	8 (18)	0.801
Asthma, n (%)	19 (17)	14 (21)	5 (11)	0.303
Diabetes, n (%)	3 (3)	0 (0)	3 (7)	n/a
Median (IQR) LCI	7.7 (6.7–8.9)	7.6 (6.8–8.6)	7.8 (6.5–9.3)	0.973
Median (IQR) CoV LCI	3.9 (2.6–6.1)	3.8 (2.8–6.1)	4.3 (2.8–6.0)	0.710
Median (IQR) CoV FRC	4.7 (2.9–7.4)	4.7 (3.1–7.7)	4.9 (2.7–6.8)	0.551
Number of visits with LCI, median (IQR)	8 (4–9)	6 (4–7)	10 (4–13)	0.007
Test time (min), median (IQR)	18.9 (15.5–22.5)	16.3 (13.5–19.3)	21.2 (18.6–24.3)	**<0.0001**

Data are shown as mean and SD if normally distributed, otherwise as median and IQR.

Data are also shown for paediatric and adult populations separately.

*Pseudomonas* status relates to that at study entry and includes patients whose status was reclassified following consent based on ongoing microbiology.

Bold values indicate statistical significance.

*P value relates to comparison of adult and paediatric values and is derived from unpaired t-test for normally distributed data, Mann-Whitney U test or two-tailed Fisher’s exact test for proportions.

BMI, body mass index; CoV, coefficient of variation; FEF_25–75_, mid-expiratory flow; FRC, functional residual capacity; LCI, lung clearance index; n/a, not applicable.

### Feasibility

Eight participants (7%) were unable to perform tests capable of producing LCI outputs: four patients after repeated attempts (including one adult) and four after attempts on a single visit. All were withdrawn or withdrew from the study. Two further subjects with equivocal sweat chloride and no CFTR gene mutations on full gene sequencing were reclassified as non-CF and excluded from all analyses. Of the 77 children consented, 70 (91%) were therefore able to perform LCI measurements, compared with 98% of adults. The remaining 112 patients completed a total of 913 LCI visits, with a median of 6 successful LCI measurements (IQR 4–9) per subject. In 67 visits, it was not possible to obtain the minimum requirement of two reproducible washout repeats, giving an overall visit success rate of 92.7%. Failed visits were more common in children (11.3% of all visits) than adults (3.3%) (p<0.001). The reasons for test failure are described in the online supplemental file.

### Patient population

In line with study objectives, patients generally had mild disease, with a mean FEV_1_ z-score of −1.2 (range −4.3 to 1.3). Nineteen (17%) had FEV_1_ >100% predicted (see also online supplemental file and figure E2). Overall 40 (36%) were pancreatic sufficient, but this proportion was significantly higher in adults (61% vs 19% in children, p=0.0001). Other indicators that adult patients were drawn from those with milder disease include a lower rate of Phe508del homozygosity (25% vs 57%, p=0.0009), older age at diagnosis (median 5.0 years vs 0.2 years, p<0.0001) and a high rate of never been infected with *P. aeruginosa* (48% vs 35%, p=0.2). There were few comorbidities overall, with only 3 (3%) patients with diabetes, 5 (4%) with CF liver disease and 19 (17%) with a coexistent diagnosis of asthma.

**Figure 2:**
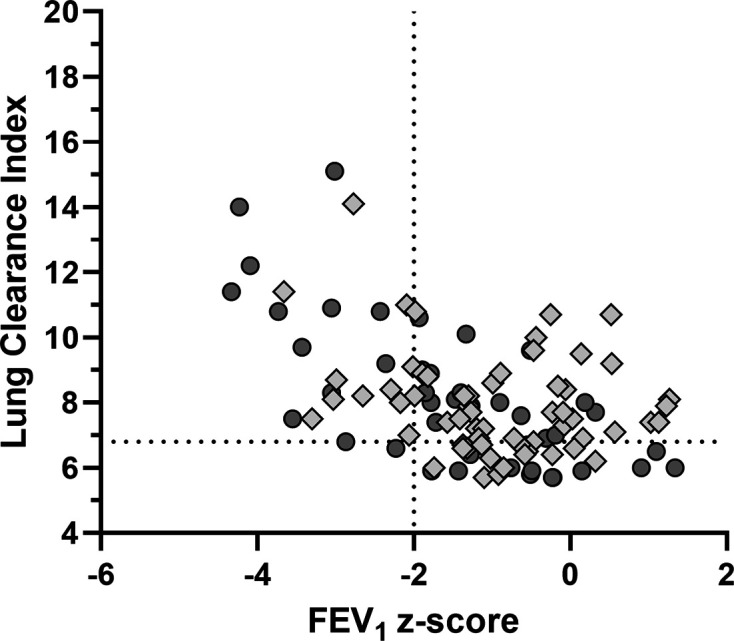
Relationship between lung clearance index (LCI) and FEV_1_ z-score in patients with cystic fibrosis at visit 1. Adult subjects are represented by dark grey circles and children by light grey diamonds. Vertical dotted line represents the lower limit of normal FEV_1_ (z-score=−2), while horizontal dotted line represents the upper limit of normal LCI (6.9). Subjects shown in the upper right quadrant have normal FEV_1_ but elevated LCI.

The median LCI at visit 1 was 7.7 (range 5.6–15.1) and there was no significant difference between children and adults (table 1). The relationship between FEV_1_ z-score and LCI at visit 1 is presented in figure 2. There was significant correlation between measurements (r=−0.43, 95% CI −0.58 to −0.25, p<0.0001). However, LCI was elevated in a large proportion of those with normal FEV_1_: of 81 subjects with normal FEV_1_ at visit 1, 51 (63%) had LCI of ≥6.9. The median total LCI test time was under 19 min, with shorter test times in children: median (IQR) 16.3 (13.5–19.3) min vs 21.2 (18.6–24.3) min in adults (p<0.0001).

### Repeatability of LCI measurements

**Figure 3:**
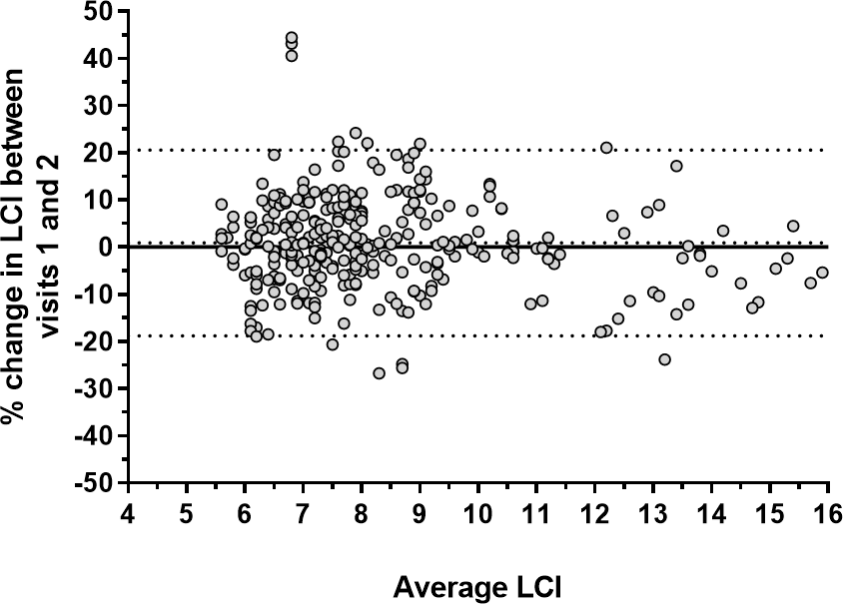
Bland-Altman plot of percent change in lung clearance index (LCI), defined as change at visit 2 compared with visit 1, against average. Central dotted line represents mean change, while outer dotted lines represent upper and lower limits of agreement.

For assessment of LCI repeatability, 80 subjects contributed 313 valid data pairs of stable LCI measurements within 6 months of each other. Of the total 781 eligible LCI measurements, 315 (40%) were excluded from repeatability assessments due to the patient being unwell or on additional antibiotics. The median (IQR) interval between included measurements was 105 (70–154) days (approximately 3.5 months). The mean (SD) absolute difference in LCI was 0.01 (0.85), representing a mean (SD) change of 0.9% (10.1) of baseline LCI. The Bland-Altman limits of agreement were therefore −18.8% to 20.7% (see figure 3 and online supplemental figure E3). The ICC was 0.93 (95% CI 0.91 to 0.94) (see online supplemental file, table E1). As a sensitivity analysis, these analyses were repeated for all unique pairs of LCI (n=152). These produced highly similar results and are presented in the online supplemental file.

### Longitudinal change in LCI

#### Latent class analysis

**Figure 4:**
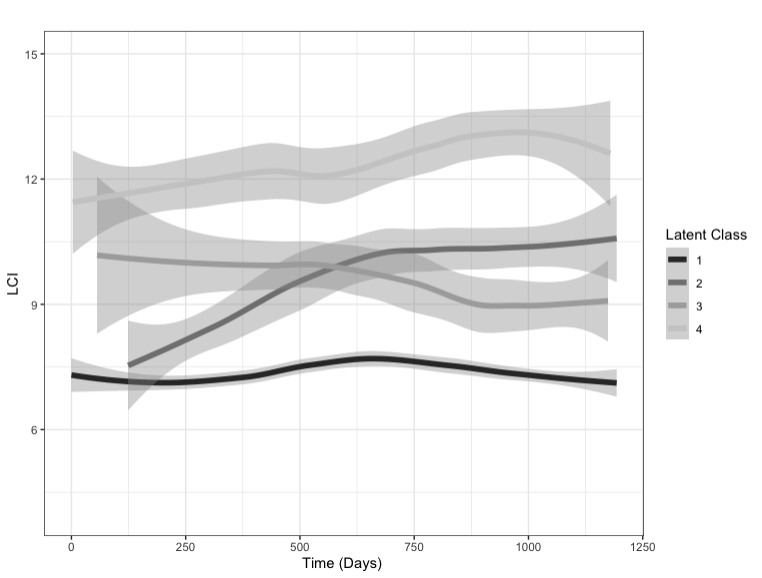
Clustering of longitudinal lung clearance index (LCI) data performed using latent class growth analysis. The graph shows the best-fit solution of four clusters, with central and grey bands representing the mean and 95% CI of the cluster trajectories. Time from first measurement is shown on the x-axis. Latent class clusters 1-4 are described in the text and in table 2. Individual patient trajectories are presented in online supplemental figure E6.

Latent class analysis identified four distinct clusters, based on the trajectory of LCI. These are shown in figure 4 and a descriptive summary is presented in table 2 with a list of clinical variables commonly used to describe CF lung disease. The four clusters were unevenly distributed, with the majority of subjects (58, 72%) being grouped together on the basis of LCI values which remained stable over the course of the study (cluster 1, ‘stable near normal’). The other three clusters consisted of low LCI rising with time (cluster 2, ‘near normal, increasing LCI’, n=8); elevated LCI falling over time (cluster 3, ‘abnormal, stable/improving’, n=7); and high LCI increasing over time (cluster 4, ‘abnormal, increasing’, n=8). There was no difference in baseline clinical variables between clusters, with the exception of baseline LCI and the spirometric indices FEV_1_, FVC and forced expiratory flow z-scores. The spirometric indices are commonly highly associated with each other, and lower spirometry values and higher LCI were found in the clusters which experienced change in LCI over time.

**Table 2:** Baseline characteristics of the four different clusters identified by latent class analysis

Baseline characteristics	All	Cluster 1 Stable, near normal LCI	Cluster 2 Near normal LCI, increasing	Cluster 3 Abnormal LCI, stable/improving	Cluster 4 Abnormal LCI, increasing	P value
n (%)	81	58 (72)	8 (10)	7 (9)	8 (10)	
Age, mean (SD)	15.5 (9.9)	14.3 (8.2)	13.8 (8.2)	20.6 (20.5)	21.6 (9.2)	0.191
Age at diagnosis, mean (SD)	4.4 (9.2)	3.6 (6.9)	3.2 (6.9)	11.3 (22.4)	5.76 (6.6)	0.221
Baseline LCI, mean (SD)	8.0 (1.7)	7.3 (0.9)	8.2 (1.4)	10.0 (0.7)	11.2 (1.5)	**<0.001**
FEV_1_ z-score, mean (SD)	−1.27 (1.30)	0.99 (1.17)	−1.34 (1.04)	−1.35 (1.15)	−3.11 (1.16)	**0.003**
FEF z-score, mean (SD)	−1.27 (1.45)	−0.99 (1.37)	−1.47 (1.30)	−1.03 (1.03)	−3.25 (0.94)	**0.0014**
FVC z-score, mean (SD)	−0.68 (1.10)	−0.46 (0.97)	−0.59 (0.86)	−1.15 (1.05)	−1.92 (1.48)	**0.031**
Total number of antibiotic courses for exacerbations/year, median (IQR)	1.2 (0.7–2.2)	1.2 (0.7–2.1)	1.6 (1.0–3.1)	1.1 (0.4–2.3)	1.4 (0.8–4.4)	0.517
Number of courses of intravenous antibiotics/year, median (IQR)	0 (0–0.4)	0 (0–0.4)	0.3 (0–1.1)	0 (0–0.5)	0.4 (0–1.3)	0.096
BMI (kg/m^2^), mean (SD)	19.5 (4.0)	19.4 (3.9)	18.6 (3.5)	19.0 (4.9)	21.7 (4.7)	0.351
Phe508del homozygotes, n (%)	41 (50.6)	30 (51.7)	4 (80.0)	4 (57.1)	3 (37.5)	0.910
Male, n (%)	45 (55.6)	31 (53.5)	3 (37.5)	6 (85.7)	5 (62.5)	0.304
Pancreatic sufficient, n (%)	23 (28.4)	17 (29.3)	1 (12.5)	2 (28.6)	3 (37.5)	0.751
*Pseudomonas aeruginosa* status, n (%)	18 (22.2)	13 (22.4)	2 (25.0)	1 (14.3)	2 (25.0)	0.991
Chronic infection with *Haemophilus influenzae*, n (%)	13 (16.5)	11 (19.6)	1 (12.5)	0 (0)	1 (12.5)	0.811
Chronic infection with *Aspergillus, n (%)*	14 (19.2)	9 (16.7)	1 (14.3)	2 (33.3)	2 (33.3)	0.472

For continuous variables, the Kruskal-Wallis test was used to compare the means of the four groups without assuming normality and homogeneity of variances. For categorical data, Fisher-Freeman-Halton exact test was used.

Bold values indicate statistical significance.

BMI, body mass index; FEF, forced expiratory flow between 25% and 75% of FVC; FVC, forced vital capacity; LCI, lung clearance index.;

LCGA was repeated for children and adults separately. Analysis is limited by sample size, but these exploratory analyses, presented in the online supplemental file, showed similar patterns of clustering into three groups (online supplemental figures E6 and E7). Also included in the online supplemental file is a sub-analysis of patients with normal-range FEV_1_ (z-score >−2) at visit 1, comparing those with normal LCI with those with high LCI (>6.9). Overall there was a greater mean change in LCI over the course of the study in those with normal FEV_1_ and normal LCI at visit 1: mean (SE) 0.60 (0.23) units vs −0.07 (0.17) units (p=0.02). However, those with high LCI were much more likely to be in one of the clusters showing change in LCI over time (29% vs 9%).

#### Linear mixed model analysis

The strongest factor associated with LCI change over time was baseline LCI (p<0.001). The model also identified age, baseline standardised FEV_1_, *Pseudomonas* status and intravenous antibiotic courses as predictors of change over time (p<0.05) (see table 3). Coefficients of the continuous covariates reflect the rate of change in LCI over time for a unit increase in that covariate. Coefficients for gender, *Pseudomonas* status and pancreatic status reflect the adjusted mean difference in LCI in the two groups over the follow-up period. Pancreatic status was added as a variable during data analysis to explore the impact of differences between child and adult populations, but was not significant and did not affect the significance of the other outcomes.

**Table 3:** Results of linear mixed model analysis to identify factors associated with change in lung clearance index (LCI)

Factor	Coefficient	SE	95% CI	P value
Time (days)	0.0002	0.0001	−0.00002 to 0.0005	0.075
LCI at baseline	0.758	0.085	0.591 to 0.925	**<0.001**
Age	0.040	0.017	0.007 to 0.073	**0.019**
Gender	−0.276	0.240	−0.746 to 0.194	0.250
BMI (kg/m^2^)	−0.028	0.040	−0.106 to 0.051	0.486
*Pseudomonas aeruginosa* status	−0.668	0.337	−1.328 to −0.009	**0.047**
FEV_1_ z-score	−0.322	0.116	−0.550 to −0.095	**0.006**
Rate of all exacerbations/year	−0.119	0.130	−0.374 to 0.135	0.357
Number of courses of intravenous antibiotics/year	0.755	0.338	0.092 to 1.417	**0.026**
Pancreatic status	−0.158	0.292	−0.731 to 0.414	0.588

The coefficient for time reflects the rate of change (per day) of LCI taking into account the effect of the other variables in the model. The remaining covariates are fixed at baseline and not time-varying, so their model coefficients represent the change in the mean LCI associated with a unit increase in that term while everything else in the model is the same. For categorical covariates such as *Pseudomonas* status, we can interpret the coefficient as an adjusted mean difference between the dichotomous groups.

*Pseudomonas aeruginosa* status: chronically or intermittently infected with *Pseudomonas aeruginosa* versus previous or never infected.

Bold values indicate statistical significance.

BMI, body mass index; FEV_1_, forced expiratory volume in 1 second; LCI, lung clearance index.

### Acceptability and clinical impact of LCI

Eighteen adult participants with CF returned LCI participant experience forms (44% of eligible adult subjects). Of the respondents, 64% identified that the test was easy to complete (see online supplemental figure E9). Using visual analogue scales, 78% of the respondents scored >80 for ease of test and 78% of respondents scored >60 for the time of test being ‘just right’ (online supplemental figure E10). When asked to identify the ‘worst part of the test’, six (30%) patients identified MBW breathing issues. These included being conscious of breathing rate (n=2), warm air from the rebreathe wash-in (n=3) and added resistance (n=1). Of the respondents, 20% did not identify any issues at all. The most frequently suggested improvement (28%) was shortening of test time, but many did not suggest any improvements (39%).

Reliable data on clinical impact were available in 27 cases (8 adult, 19 paediatric). Individual LCIs are presented against impact in online supplemental figure E11. In 48% of cases LCI had no impact on clinical decision-making (ie, in concordance with clinical and lung function data). In 30% of cases LCI was identified as having a ‘partial impact’ on outcome. In the remaining five cases (19%) LCI was rated as having a ‘strong impact’ on clinical outcome, including prescription of additional antibiotics in four of these cases.

## Discussion

In this multicentre, prospective study we have successfully introduced routine LCI measurements into clinical practice in children and adults. This has enabled the measurement of repeatability and change over time and identified different patient clusters based on LCI and trajectory of LCI. *Pseudomonas* infection is known to be associated with higher LCI values, so this study specifically targeted those free of chronic infection and with generally mild impairment in FEV_1_. They may not therefore be representative of the entire CF population, which may explain why less change in LCI over time was seen than in studies with a greater proportion of *Pseudomonas*-infected patients.^24 25^ With well-preserved lung function increasingly common in older children and adults, and likely to be more so with CFTR modulator therapies, there is however a greater unmet need for clinically scalable sensitive lung function monitoring in this group of subjects and for knowledge of how this evolves over time.

A challenge of assessing stability of LCI in CF is that ventilation heterogeneity is an inherently unstable property due to shifting patterns of mucus that can change with physiotherapy and treatments as well as underlying disease state.^10 26^ Thus although within-session repeatability of LCI was good and similar to that of healthy controls,^10^ the between-session repeatability over 6 months was ±20%. This is narrower than that previously reported in preschool children^27^ and similar to that of school-aged children with CF.^28^ Tighter reproducibility may be seen over shorter time spans or in clinical trials, but for clinical practice this figure therefore seems relatively robust across different age groups and devices.

More relevant to clinical practice than simple paired measurements, we have also described longitudinal trajectories of LCI to determine risk factors for progressive disease. It was reassuring that the majority of these patients with predominantly mild CF fell into a cohort with stable LCI throughout the course of the study. Similar findings were reported recently in school-aged children.^29^ This has inevitably also made it harder to detect significant differences between the remaining cohorts. Nonetheless, 10% of participants showed evidence of LCI progression from a low (ie, well-preserved) baseline, and a similar proportion showed LCI rising from a higher baseline. Using linear mixed model analysis we identified age, baseline LCI, baseline FEV_1_, *Pseudomonas* status and rate of exacerbations requiring intravenous antibiotics as differentiating factors. This matches observations in patients with more severe disease that those with lower lung function, older age and with chronic *Pseudomonas* are more likely to show longitudinal decline in lung function.^25 30^ Using these longitudinal data, we have also assessed the point-of-care impact of contemporaneous LCI results on clinical decision-making. This was challenging to deliver, requiring both pre-preparation of reports and rapid analysis and integration of real-time data. For these reasons numbers are limited, but in over half of cases clinicians identified that the measurement provided additional information about clinical status, above that from clinical review and spirometry.

There have been a small number of other longitudinal LCI studies, and a common picture of LCI in monitoring CF is emerging. For very young children, elevated preschool LCI seems to predict higher LCI at early school age^29^ and at adolescence.^12^ LCI seems to remain relatively stable during the early school years,^29 31^ but increases in adolescence,^31^ a time when predicted FEV_1_ may be less useful due to rapid changes in lung size. Steeper changes in LCI over time are also seen with concurrent *Pseudomonas* infection.^31^ It has been recognised for some time that better tools to monitor lung function decline in CF are required,^32^ and on the face of it LCI fits this bill well. There remain however significant challenges relating to the delivery of this measurement in routine practice, and this has so far only been successfully delivered in a handful of institutes. Barriers to routine use include technical, training and clinical factors. From a technical perspective, a range of devices are now available, requiring differing techniques and demonstrating poor correlation across systems,^19 33–35^ which has probably hindered clinical integration.

We addressed the practical challenges by using a system with closed-circuit wash-in, which allowed rapid, portable measurements and reduced total test time to a median of around 20 min. MBW assessment was well tolerated by patients, with no major or consistent issues identified with the procedure itself, although the biggest single user complaint remained one of time taken to complete testing. Delivering quality-controlled measurements to clinicians required real-time review and analysis by an experienced operator and may not be feasible in all clinics. What we have shown is that for the majority of patients with mild CF LCI remained stable. For most CF centres struggling with the practicalities of delivering real-time MBW, a more realistic and practical ambition would be to perform these less frequently, for example at annual review or on request (eg, where spirometry is equivocal or unreliable). Based on our observations, this would help to identify those with low and stable LCI as well as those with raised or progressively deteriorating LCI who would merit further assessment or treatment. This addresses one of the CF monitoring challenges recently posed by the UK National Institute for Health and Care Excellence.^15^ A combination of borderline-raised LCI, with or without impaired FEV_1_, and increased need for rescue antibiotics may represent a group to target in future trials to investigate whether LCI trends can be reversed.

Delivery of LCI measurements in real-world clinical setting is both a strength and a limitation of this study. Intervals between visits were not standardised, with adult patients in particular being assessed more frequently than every 3 months. This has led to varying numbers of assessments for different subjects. On the other hand, this study was focused on assessing the value of LCI in a clinically relevant population (those with mild disease) and in a clinically realistic setting, where assessments may not be rigidly scheduled. Adult patients in this study appeared to be phenotypically somewhat different from the children, with a lower proportion with ‘classical’ CFTR mutations and pancreatic insufficiency. In order to resolve this, analyses were repeated for the adult and paediatric populations separately. Small numbers in some cohorts mean that these additional analyses should be considered as exploratory only.

In summary, we have shown that, with the appropriate resources, LCI can be routinely delivered in a clinical setting and is generally acceptable to patients. Most patients with CF with well-preserved lung function show stable LCI over time; however, cluster behaviours can be identified that could serve as interventional groups in future studies. We have reported on acceptable repeatability of clinical measurements and shown that baseline LCI is a risk factor for future progression of LCI. These results support the use of LCI in clinical practice in identifying patients at risk of lung function decline, but the measurement is challenging to deliver in routine practice and in many cases may be better suited to annual assessments.

## Data Availability

Data are available upon reasonable request to the corresponding author.
