# Supplementary material for: Longitudinal assessment of lung clearance index to monitor disease progression in children and adults with cystic fibrosis
Source: Thorax. 2021 Jul 22;77(4):357–63. doi: 10.1136/thoraxjnl-2021-216928 (PMC8938654; doi:10.1136/thoraxjnl-2021-216928)
Supplement: Supplementary data [file thoraxjnl-2021-216928supp001.pdf]

Longitudinal assessment of lung clearance index to monitor disease progression in children and adults with cystic fibrosis

Alex R. Horsley, John Belcher, Katie J. Bayfield, Brooke G. Bianco, Steve Cunningham, Catherine L. Fullwood, Andrew M. Jones, Anna Shawcross, Jaclyn A. Smith, Anirban Maitra, Francis J Gilchrist

Online Data Supplement to https://doi.org/thoraxjnl-2021-216928

Additional Methods

1. *Clinical Data*

Use of additional antibiotics were recorded at each patient visit. A physician-diagnosed exacerbation was defined as additional antibiotics prescribed for a change in respiratory symptoms. Courses of consecutive antibiotics, without an antibiotic-free interval, were scored as a single exacerbation. Mixed use of oral and intravenous (IV) antibiotics (either simultaneously or sequentially) were scored as an exacerbation requiring IV antibiotics, regardless of IV course length. Exacerbations were expressed as events per year, based on the patient-specific length of follow-up.

This study took place before the widespread introduction of CFTR modulator therapies. One adult and three paediatric patients with class III gating mutations were on Ivacaftor. Three patients took part in blinded trials: one withdrew to do this; the other two adult patients remained in the study but their data during the blinded phase have not been used.

2. *Multiple Breath Washout (MBW)*

MBW was performed using a closed circuit Innocor™ system (PulmoTrace ApS, Glamsberg, Denmark), as previously described (1, 2). Wash-in was performed from a sealed bag filled with a mixture of room air and test gas (94% O_2, 1% SF_6 and 5% N_2O) up to a total bag volume of 4L, adjusted according to patient size. Expired air was depleted of CO_2 prior to re-inspiration. At the start of wash-in, participants took 5-6 slow deep inhalations before returning to tidal breathing. Final washin concentration of expired SF_6 was between approximately 0.1 and 0.4%, depending on the starting concentration in the bag and the ratio of bag volume to FRC, as previously described (2). At the end of wash-in, the

Horsley AR, *et al. Thorax* 2022; 77:357–363. doi: 10.1136/thoraxjnl-2021-216928

participants were switched to room air using fast-responding pneumatic valves within the breathing unit, and instructed to maintain tidal breathing. Washout was continued until expired end-tidal SF_6 concentration reached <2.5% of the starting concentration. There was no requirement for a delay between end of washout and start of next wash-in, and subjects started the next test as soon as they were able. Distraction was provided by showing age-appropriate movies or TV shows. In the case of adults, visual feedback of inspiratory volumes was also available to aid reproducibility of breathing patterns, and typically set at 10-15ml/kg. Washouts were performed in the outpatient clinic rooms or on the ward using a portable system as previously described(2).

Both children and adults used identical patient interfaces and mouthpieces, with the only difference being that a smaller filter was used in children (subjects <18yrs). Due to refinements in the patient interface over the 3 years of the study, total deadspace varied from 50 to 58mls for the paediatric setup and from 55-65mls for the adult setup. Children transitioning to adult care also transitioned from paediatric to adult setup. It has been assumed that these small changes in deadspace volume have not affected measurements.

Subjects completed three washouts. If one or more tests were obviously compromised (e.g. evidence of leak), then additional tests were added. Detailed analysis and quality control were performed in a separate offline custom-built washout analysis package prepared in Igor Pro v6 (Wavemetrics Inc., Lake Oswego, OR, USA), as previously described (2-5). Washout repeats were excluded if there was evidence of leak, or in case of large differences seen in LCI or FRC measurements (>25% from median) (6). Final LCI and FRC measurements quoted are the average of at least two reproducible repeats. Operator training and quality control was led by the study lead (AH). Completed test files were sent electronically for centralised review by AH, who also analysed all washouts.

Washout test time was taken from the length of the washout file. This is the total time to complete all wash-in and washout tests, including additional tests required, any interval between tests, and analyser warm-up time (60 seconds). It does not include time taken to explain the test to the participants, or time taken to clean the apparatus between volunteers.

3. *Patient experience questionnaire*

Adult study participants were asked to complete a participant experience form immediately after testing, on a single occasion in the final 12 months of the study. The form provided opportunity for free-text feedback about the MBW test, asked subjects to identify the worst part, and provided visual analogue scales (VAS) out of 100 on

- “How easy was the test was to perform?” (“Not at all easy” to “Very easy”)
- “Rate the time taken to complete” (“Far too long” to “Just right”).

4. *Assessment of clinical impact*

For the final 6 months of the study period, clinicians were asked to rate the impact of LCI on clinical decision making. Patient data were loaded into the study database which was used to generate graphic reports showing all stored LCI and spirometry to date, along with times of exacerbation marked on the graph. Clinicians were provided with training to understand LCI and data on LCI variability generated in the first half of the study period. Assessment of impact depended upon having completed the LCI measurement before clinical review (in some cases not possible due to logistic issues) and having the data analysed and QC-checked before clinical review, which required the presence of an experienced operator (AH). Providing these conditions were met, the physician reviewing the patient recorded the clinical outcome immediately after reviewing the patient and rated the impact of the LCI measurement on that decision process as below:

1 – None. LCI data not relevant to clinical decision/outcome.

2 – Partial. LCI data played some role in clinical decision/outcome.

3 – Strong. LCI data were major factor in clinical decision/outcome.

Reasons for no impact could include all data in concordance (e.g. patient clinically stable with no change in LCI or other lung function measures) or patient clearly unwell and likely to receive treatment irrespective of LCI.

5. *Statistical analyses*

Data were analysed using Prism version 8 (GraphPad, San Diego, USA), R version 3.6.0 (Vienna, Austria) and Stata version 15.1 (IBM, New York, USA). Parametric data were expressed as mean (standard deviation) and nonparametric data expressed as median (interquartile range). Comparisons were performed using unpaired t test for normally distributed data, Mann-Whitney U test, or 2-tailed Fisher's exact test for proportions. No adjustment was made for multiplicity and $p<0.05$ was considered statistically significant.

Target population size was 70 patients with regular follow-up, estimated to provide sufficient numbers for robust longitudinal monitoring. There was no formal power calculation and over-recruitment was permitted.

6. *Latent class growth analysis*

Latent class growth analysis (LCGA) is a person-centred method which can be considered as a special type of latent variable modelling(7-9). LCGA models allocate individuals into different groups or classes based on the shape of their latent growth curve trajectory(9). Thus each class is summarised

by a latent growth curve with an estimated mean intercept and mean slope. The class's intercept and slope are referred to as "latent parameters" since these parameters were unobserved prior to undertaking the analysis(10). In LCGA, the variance and covariance within each latent class is eliminated by fixing the variance of the intercept and slope to zero(7, 9, 10). Due to this lack of within-class variance, LCGA models therefore assume that all individual growth trajectories within a specific class are homogenous(7, 8, 10). LCGA can thus be thought of as a fixed effect model(11). This means that all class members have the same intercept, linear slope and quadratic slope(7, 10). Individuals are probabilistically assigned to the latent class which best reflects their latent trajectory; with individuals assigned to the class for which they have the highest posterior probability(12). The latent class growth analysis process sequentially increases the number of latent classes, until the optimal number of classes is determined (10).

Latent class growth analysis (LCGA) was used to identify distinct trajectories of LCI data, enabling classes of people with similar trajectories to be identified. LCGA was undertaken using the LCMM package in R using full information maximum likelihood (FIML). After fitting a one-class quadratic LCGA model, the number of trajectory classes was increased sequentially. The statistical fit of each model was assessed by comparing Bayesian Information Criteria (BIC), Akaike Information Criteria (AIC), Size Adjusted BIC (SABIC), Entropy and the number of members per class (%) based on the most likely class membership. Lower values for BIC, AIC and SABIC indicate a better fitting model. Cluster sizes smaller than 1% of the total cohort are considered to be insufficient while entropy cut-offs of 1.0 (perfect), 0.8 (high), 0.6 (medium), and 0.4 (low) have been proposed(13).

After determining the optimal number of trajectory classes, the baseline LCI-specific characteristics of each trajectory class were compared descriptively using Stata version 15.1 and modelled by weighted multinomial logistic regression, the weights reflecting the uncertainty in estimating the latent cluster membership.

Supplementary Results

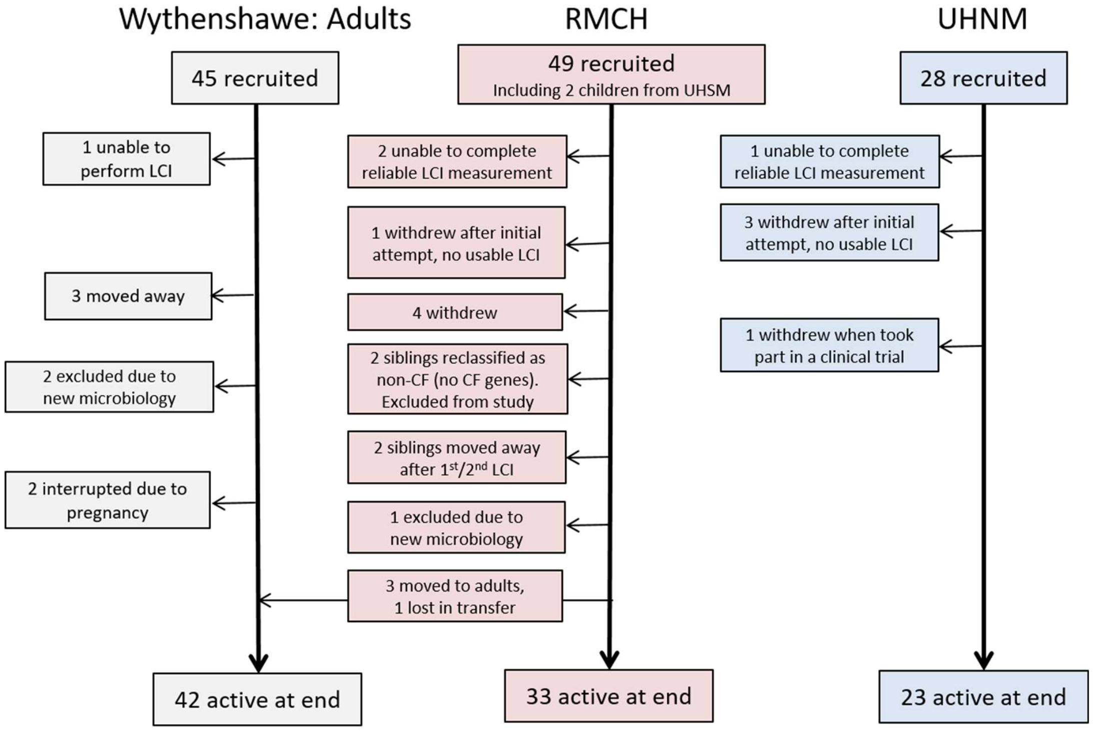

Figure E1: Consort diagram showing outcomes of adults and children with CF recruited to a longitudinal study of LCI measurements. Patients are shown in groups according to the site at which they were recruited. RMCH: Royal Manchester Children's Hospital; UHNM: University Hospital North Midlands.

Horsley AR, *et al. Thorax* 2022; 77:357–363. doi: 10.1136/thoraxjnl-2021-216928

Feasibility of LCI

Excluding those visits where LCI was not attempted, and excluding also the patients described in the main manuscript who were unable to perform LCI, there were 846 LCI measurements made on 112 subjects. In addition to this, there were another 6 visits where only a single usable washout repeat was obtained, and 61 visits where no usable repeat washouts were completed. These 67 failed assessments represented 7.3% of all visits where washout was attempted, giving a success rate of 92.7%.

Failed assessments were more common in children (52/462 visits, 11.3%) than adults (15/451 visits, 3.3%), $p<0.001$. Reasons for failure to obtain quality controlled washouts included patient-related issues such as inability to concentrate and complete a washout test (n=3, 4.5% all test failures). At another 22 visits (32.8%), test failure was due to washout technique issues (such as incomplete washin or washout, excessive breath volumes) that had not been successfully corrected at the time of testing. In 2 cases (3.0%), washout repeats were not reproducible enough to combine. The most common causes of washout failure however, accounting for 40 visits (59.7% of all failed visits), were technical issues relating to the washout system. Some of these were easily corrected, whilst one LCI machine in particular had a leaking valve which took longer to correct and resulted in the loss of several washout datasets.

Of 846 successful LCI visits, a full set of triplicate LCI repeats was available for 683 assessments (81%). 163 repeats were excluded, making up 6.4% of the total. The usual reasons for excluding a repeat were due to poor reproducibility or due to a washout not meeting quality control (eg inadequate washin, air leak). These data, and data on how many visits required a fourth washout to obtain a triplicate dataset, were not captured separately. Operators were encouraged to include a fourth washout if they suspected quality control was poor. Total test time includes all attempted washout repeats, whether included or not

Baseline FEV_1

In the original protocol, mild disease was defined as those with best FEV_1 in last 6 months as >60% predicted. This lower limit was subsequently reduced in order to capture those with recent dips in FEV_1, an issue identified in some adults. At visit 1, some patients were additionally unable to achieve their recent best spirometry. Overall, 6 adults (14%) and 2 children (3%) had an FEV_1 below 60% predicted at visit 1, whilst 25 adults (57%) and 46 children (70%) had FEV_1 above 80% predicted. Distribution of FEV_1 at visit 1 is shown in Figure E2.

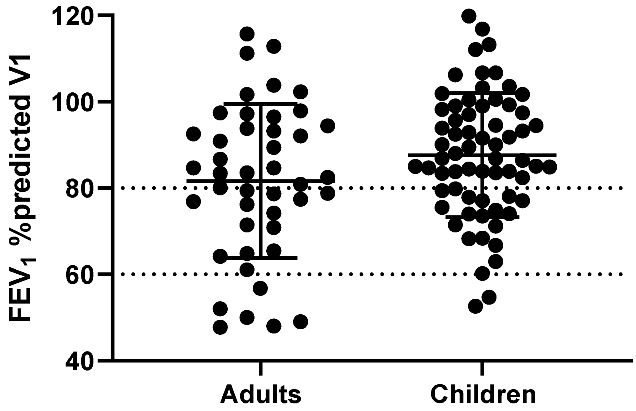

Figure E2: Distribution of FEV_1 as percent predicted, measured at visit 1. Overall, 57% of adults and 70% of children had FEV_1 within the normal range. Horizontal dotted lines show FEV_1 80% predicted (above which FEV_1 may be considered within normal range) and FEV_1 60% predicted (above which is considered mild impairment in FEV_1).

Horsley AR, *et al. Thorax* 2022; 77:357–363. doi: 10.1136/thoraxjnl-2021-216928

Repeatability

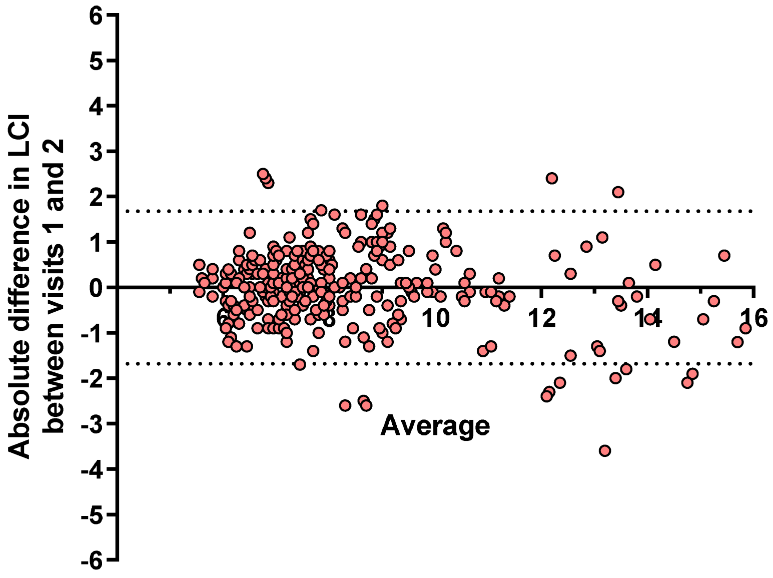

Figure E3: Bland-Altman plot of **absolute** change in lung clearance index (LCI), defined as change at visit 2 compared to visit 1, against average. Central dotted line represents mean change, outer dotted lines represent upper and lower limits of agreement.

Category	Summary Values Mean (sd)	Bland-Altman Limits of Agreement	Coefficient Variation (%)	ICC (95% CI)	Coefficient of Repeatability
**Overall**					
v1-v2	0.02 (0.82)	-1.58, 1.63	2.69	0.93 (0.91,0.94)	2.27
(v1-v2)/v1 (%)	1.05 (9.76)	-18.08, 20.18	10.26		
**Children**					
v1-v2	-0.02 (0.85)	-1.68, 1.64	2.72	0.95 (0.93,0.96)	2.35
(v1-v2)/v1 (%)	0.56 (9.98)	-18.99, 20.12	5.63		
**Adults**					
v1-v2	0.10 (0.73)	-1.33, 1.54	13.95	0.81 (0.71, 0.88)	2.03
(v1-v2)/v1 (%)	1.99 (9.12)	15.89, 19.87	21.78		

Table E1: Repeatability of lung clearance index (LCI). Four methods of assessing repeatability of LCI are presented: Bland-Altman limits of agreement, coefficient of variation (%), Intraclass Correlation Coefficient (ICC), and coefficient of repeatability. Data are presented for the combined dataset and for adults and children separately. Change in LCI is expressed both as absolute change in LCI between visits (V1-V2) and as percent change ((V1-V2)/V1). ICC was only calculated for absolute change in LCI.

Sensitivity analyses

Repeatability analyses were also conducted using only unique pairs of LCI, ie where both data points were only used in the first pairing in which they occurred. This resulted in 152 valid data pairs, with a median (IQR) interval between measurements of 91 (61-126) days. Mean (SD) absolute difference in LCI was 0.06 (0.84). Mean (SD) percent change in LCI was 1.6 (9.8), making the Bland-Altman limits of agreement -17.6 to 20.7%. These data are shown in Figures E4 and E5.

Analyses were also conducted for adults and children separately. The adults contributed 208 of the data pairs (66%). Median % difference in LCI at visit 2 was -0.5%, whilst for children the median % difference in LCI at visit 2 was 1.9%, p=0.015.

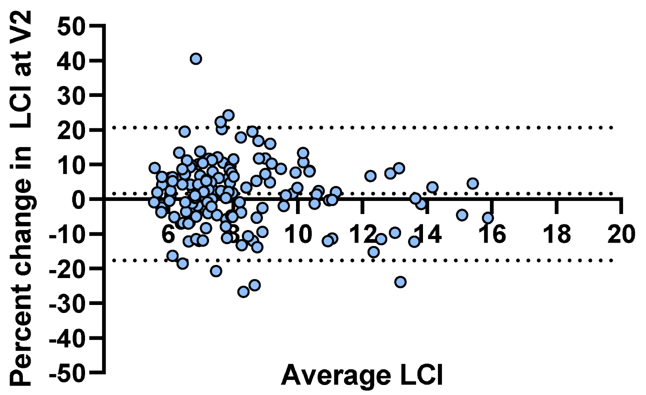

Figure E4: Bland-Altman plot of percent change in lung clearance index (LCI), defined as change at visit 2 compared to visit 1, against average, for unique data pairs only. Central dotted line represents mean change, outer dotted lines represent upper and lower limits of agreement.

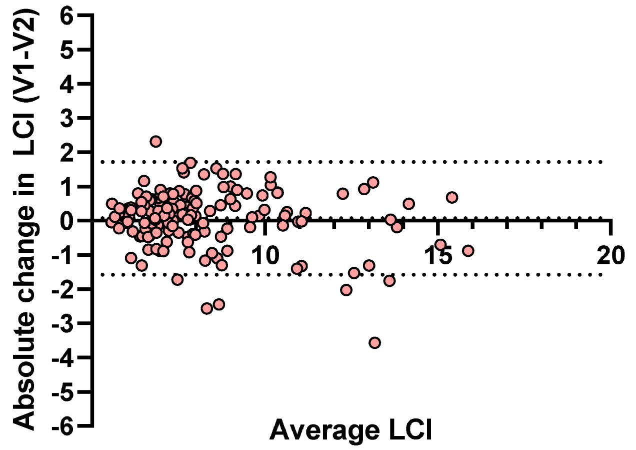

Figure E5: Bland-Altman plot of absolute change in lung clearance index (LCI), defined as change at visit 2 compared to visit 1, against average for unique pairs of data only. Central dotted line represents mean change, outer dotted lines represent upper and lower limits of agreement.

Latent Class Growth Analysis

The 4-cluster solution was considered the best fit since it returned the lowest BIC, SABIC, AIC and yielded high entropy (Table E2). All class sizes were>1% of the total number of participants. The degree to which the trajectory classes captured distinct and important patterns in the data was assessed by estimating the average posterior probability for each cluster. These values are presented for the 4-cluster solution in Table E3 and follow the GRoLTS-Checklist (14). Individual and mean LCI trajectories for the 4-cluster solution for the combined dataset are shown in Figure E6, and described in the main manuscript text.

These analyses were repeated for the adults (n=29) and children (n=52) separately and are presented in Figures E6 and E7. Due to the smaller numbers of subjects in each of these cohorts, these analyses should be considered as exploratory only. In both cases the cohorts clustered into 3 groups; there were insufficient data to form the same four groups seen with the full dataset. Also in both cases the largest group was those with stable LCI. Posterior probabilities were high for almost all clusters (>0.9). In both adults and children, univariate modelling identified differences in baseline LCI across cohorts (P<0.0001). Differences in FEV_1 across the cohorts were only seen with the adult data (p=0.008). No other factors were significantly associated with clusters.

Cluster Number	Loglik	AIC	BIC	SABIC	entropy
1	-862.958	1735.916	1747.888	1732.12	1
2	-852.0611	1720.122	1739.278	1714.048	0.8685657
3	-845.2793	1712.559	1738.898	1704.207	0.8560668
4	-837.1697	1702.339	1735.862	1691.71	0.877377
5	-837.1697	1708.339	1749.045	1695.433	0.6253255

Table E2: **Model fit results for latent class growth analysis of lung clearance index trajectories**. Optimal fitting and entropy are shown for the model using 4 clusters. Loglik: log likelihood. BIC: Bayesian Information Criteria. AIC: Akaike Information Criteria. SABIC: Size Adjusted BIC.

Horsley AR, *et al. Thorax* 2022; 77:357–363. doi: 10.1136/thoraxjnl-2021-216928

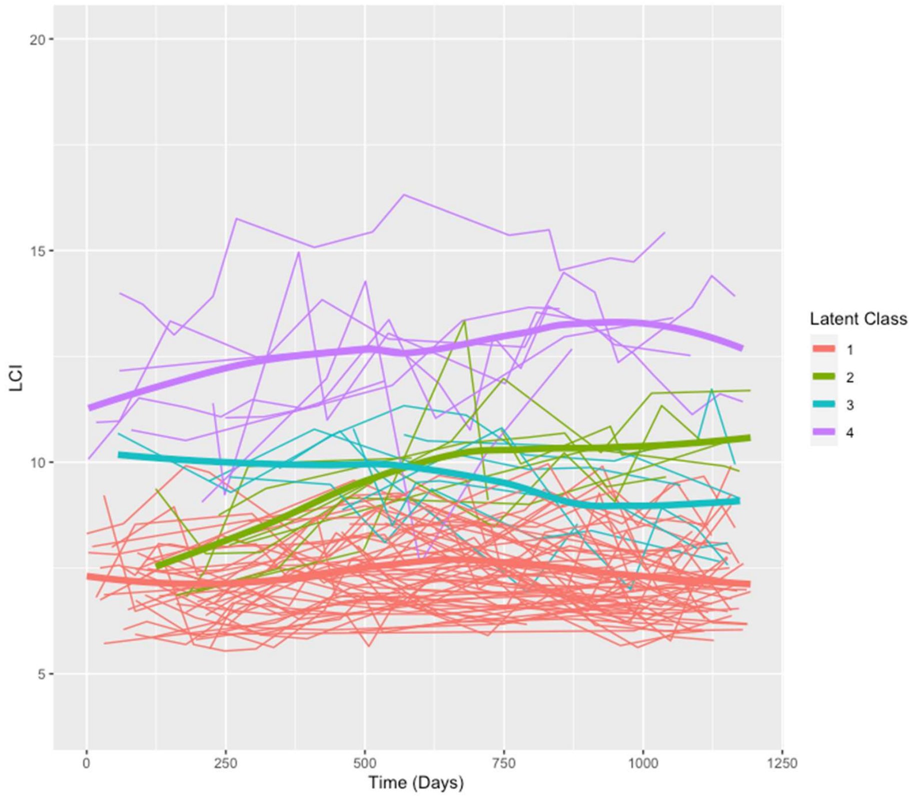

Figure E6: Clustering of longitudinal lung clearance index (LCI) data for the whole dataset performed using latent class growth analysis. Each individual patient's LCI data are represented by a single line, with time from first measurement shown in days on the x axis. Data are clustered around four discrete trajectories, with individual profiles shown in the colour of the class they are clustered with. Clusters are described in the text of the main manuscript and in Table E3, the posterior classification table (below).

	Description	N (%)	Probability 1	Probability 2	Probability 3	Probability 4
**Class 1**	Stable, near normal LCI	58 (72%)	0.9484	0.0198	0.0318	0.0000
**Class 2**	Near normal LCI, increasing	8 (10%)	0.0388	0.9051	0.0541	0.0020
**Class 3**	Abnormal LCI, stable/ improving	7 (9%)	0.0744	0.0136	0.9006	0.0114
**Class 4**	Abnormal LCI, increasing	8 (10%)	0.0000	0.0446	0.0218	0.9336

Table E3: Posterior Classification table for the 4-cluster LCGA model: average posterior probability for each trajectory class, representing the mean probability of an individual having that cluster assignment given their observed data.

Horsley AR, *et al. Thorax* 2022; 77:357–363. doi: 10.1136/thoraxjnl-2021-216928

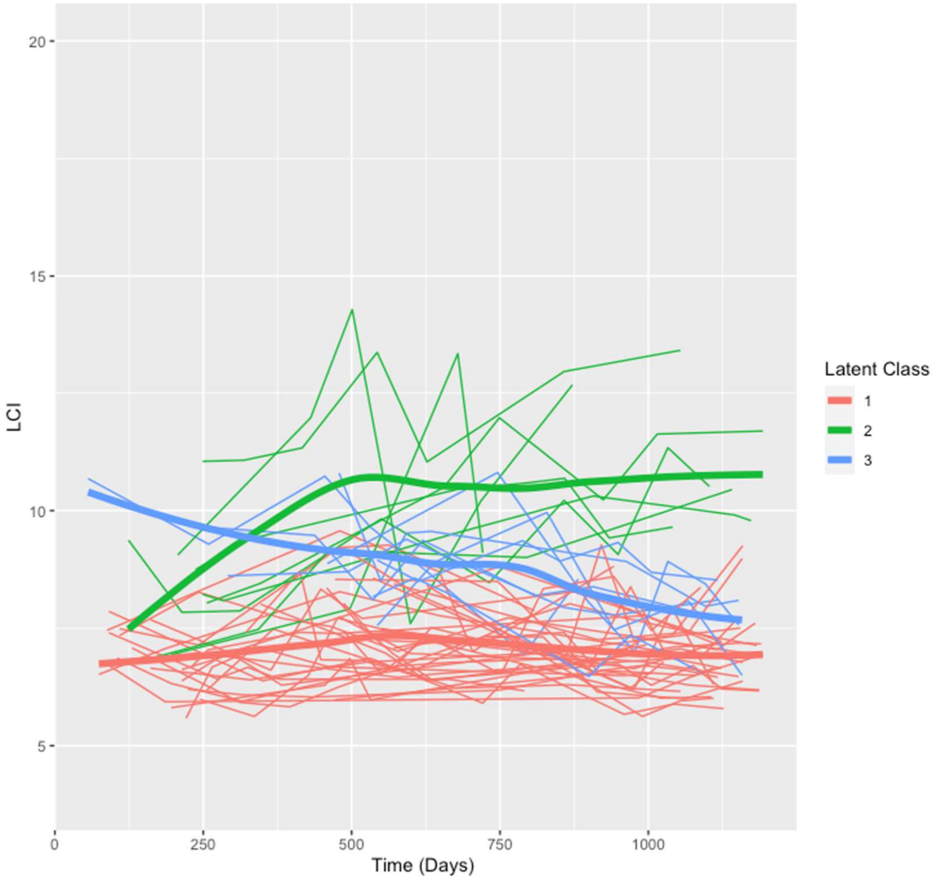

Figure E7: Clustering of longitudinal LCI data for the paediatric cohort only, performed using latent class growth analysis. Each individual patient's LCI data are represented by a single line, with time from first measurement shown in days on the x axis. Posterior classification table is presented below, representing the mean probability of an individual having that cluster assignment given their observed data.

	Description	N (%)	Probability 1	Probability 2	Probability 3
**Class 1**	Stable LCI	36 (69%)	0.9977	0.0019	0.0004
**Class 2**	Rising LCI	8 (15%)	0.0076	0.9409	0.0515
**Class 3**	Falling LCI	8 (15%)	0.0046	0.2286	0.7668

Table E4: Posterior Classification table for the 3-cluster LCGA model for paediatric lung clearance index data: average posterior probability for each trajectory class

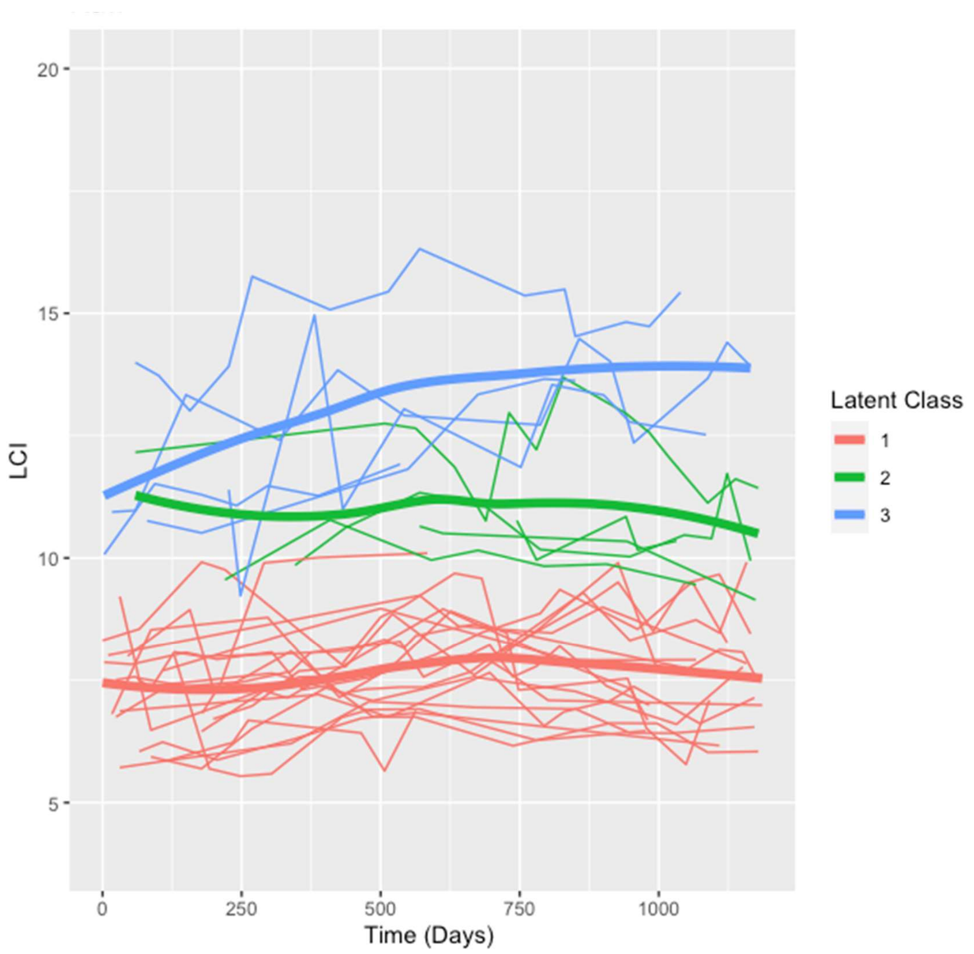

Figure E8: Clustering of longitudinal LCI data for the adult cohort only, performed using latent class growth analysis. Each individual patient's LCI data are represented by a single line, with time from first measurement shown in days on the x-axis. Posterior classification table is presented below, representing the mean probability of an individual having that cluster assignment given their observed data.

	Description	N (%)	Probability 1	Probability 2	Probability 3
**Class 1**	Stable LCI	19 (65%)	0.9933	0.0065	0.0002
**Class 2**	High stable LCI	5 (17%)	0.0576	0.9400	0.0024
**Class 3**	Rising LCI	5 (17%)	0.0001	0.0717	0.9282

Table E5: Posterior Classification table for the 3-cluster LCGA model for adult lung clearance index data: average posterior probability for each trajectory class

Horsley AR, *et al. Thorax* 2022; 77:357–363. doi: 10.1136/thoraxjnl-2021-216928

Trajectory based on initial FEV_1 and LCI

The group with normal-range FEV_1 (defined as z score >-2) but raised LCI were investigated further, and compared to those with normal FEV_1 and normal LCI. Such patients represent a group collectively identified as "normal" by spirometry but divided here by LCI to explore whether this measurement, at a single visit, could provide insight into future outcomes. This analysis was performed post-hoc, and was not a part of the original analysis plan.

This analysis was only conducted on those included in the longitudinal dataset. Of these, 63 patients had normal FEV_1 at their first visit, of whom 41 (65%) had elevated LCI (>6.9). Visit 1, LCI trajectory, and cluster distribution are shown below for these two groups.

Group description	Normal FEV_1, normal LCI	Normal FEV_1, raised LCI	P value
**n**	22	41	
**Mean (SD) FEV_1 z score**	-0.70 (0.77)	-0.63 (0.89)	0.7
**Median (IQR) LCI**	6.49 (5.99-6.77)	7.86 (7.42 - 8.91)	<0.001
**Mean (SE) group change in absolute LCI**	0.60 (0.23)	-0.07 (0.17)	0.02
**Mean rate of change per year in LCI (SE)**	0.082 (0.076)	0.032 (0.091)	0.70
**Cluster 1 (%)**	20 (91)	29 (71)	
**Cluster 2 (%)**	2 (9)	4 (10)	
**Cluster 3 (%)**	0	6 (14.6)	
**Cluster 4 (%)**	0	2 (4.9)	

Table E6: Comparison of those in the longitudinal analysis who had normal-range FEV_1 at visit 1, divided by their LCI at visit 1. Clusters refer to the clusters identified in latent class growth analysis and described above (Table E3).

There was no difference in baseline FEV_1 between the groups. Median LCI was, inevitably, significantly higher in the group with raised LCI. Mean absolute change in LCI over the course of the study in the group with high LCI was close to zero, and the mean slope was not significantly non-zero. In the group with normal LCI, there was a significantly higher mean change in absolute LCI over the study of 0.6 units, but mean slope was not significantly non-zero. However there were also differences in cluster distribution, with almost all (91%) of those with normal LCI being in cluster 1, indicating stable LCI over time. Patients with elevated LCI and normal FEV_1 were more likely to be in one of the other clusters

(29%) indicating change in LCI outcomes over time. Positive and negative changes in LCI (clusters 2 and 4, vs cluster 3) were equally likely, leading to an overall minimal change in mean LCI.

Participant experience questionnaires

Questionnaires were given to participants at the time of LCI review and handed in separately to the clinic nurse. Five previously-recruited subjects were not seen during the questionnaire period, making the eligible population 41 adult subjects (including those transitioned to adult care from paediatrics). Eighteen completed questionnaires were received (44% eligible population). Responses to free text were grouped into categories. Visual analogue scores (VAS) were taken from measurements of the point where the mark made by the patient crossed the score line, and are shown graphically in bins of 10mm (Figure E10).

Question 1: "How did you find the washout testing?"

32% identified that they had experienced no problems and a further 32% answered "ok". Some added additional comments to say the test required little effort or time (n=3), one indicated that the first test had been the hardest, and one that it was harder when unwell. One respondent answered that the test was "long" and another that it was "boring".

Question 2: What was the worst part of the test?

Responses to this question are shown in below in Figure E9.

Question 3: How could the test be improved?

7/18 respondents (39%) were unable to identify any ways to improve the test. Five (28%) felt the test time was too long, one that the apparatus needed to provide more leg room, two recommended more practice before starting testing. Three subjects felt there should be a better selection of films provided for distraction during tidal breathing.

Horsley AR, *et al. Thorax* 2022; 77:357–363. doi: 10.1136/thoraxjnl-2021-216928

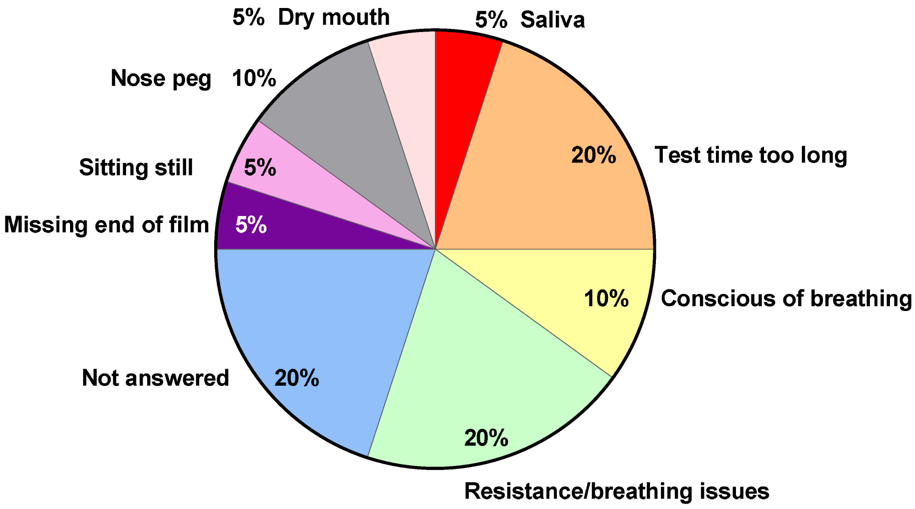

Figure E9: Responses by participants to the question "What was the worst part of the [MBW] test?" Responses were free text and have been grouped into themes. Participants could identify more than one issue. Total number of separate items = 20 from 18 respondents.

Question 4 (VAS): How easy did you find it to complete test?

Participants were asked to mark their experience on a 100mm VAS from "Not at all easy" to "Very easy". Responses are shown in Figure E10. 78% scored over >80mm for ease of testing.

Question 5 (VAS): 5. How do you rate the time taken to complete one set of tests?

Participants were asked to mark their experience on a 100mm VAS from "Far too long" to "Just right". Responses are shown in Figure E10. 78% scored over >60mm for "Just right".

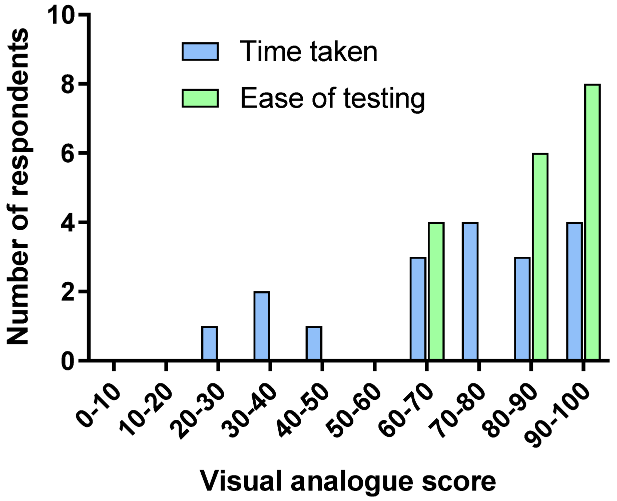

Figure E10: Summary of individual participant responses to two visual analogue score questions on their experience of multiple breath washout testing. Scores were measured and placed into 10mm bins. Ease of testing (shown in green) was scored from 0mm – “Not at all easy” to 100mm - ”Very easy”. Time taken (shown in blue) was scored from 0mm – “Far too long” to 100mm - ”Just right”.

Impact

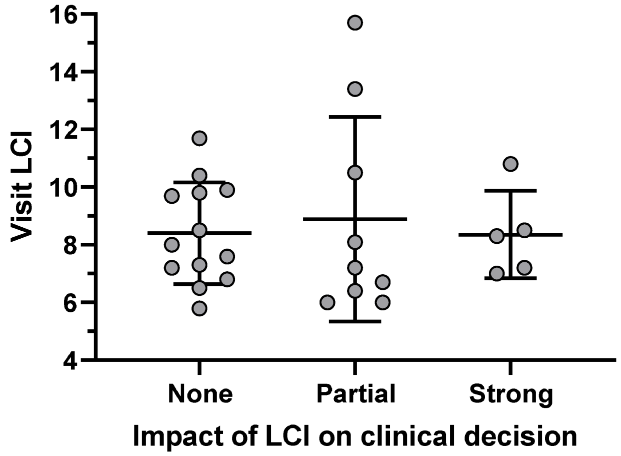

Figure E11: Comparison of visit LCI and the clinician-rated impact of the measurement on the clinical decision making. There were no statistically significant differences between the groups (p=0.9)

References

1. Horsley AR, O'Neill K, Downey DG, Elborn JS, Bell NJ, Smith J, et al. Closed circuit rebreathing to achieve inert gas wash-in for multiple breath wash-out. ERJ Open Research. 2016;2(1).
2. Horsley AR, Alrumuh A, Bianco B, Bayfield K, Tomlinson J, Jones A, et al. Lung clearance index in healthy volunteers, measured using a novel portable system with a closed circuit wash-in. PLoS One. 2020;15(2):e0229300.
3. Davies J, Sheridan H, Bell N, Cunningham S, Davis SD, Elborn JS, et al. Assessment of clinical response to ivacaftor with lung clearance index in cystic fibrosis patients with a G551D-CFTR mutation and preserved spirometry: a randomised controlled trial. Lancet Respir Med. 2013;1(8):630-8.
4. Bell AS, Lawrence PJ, Singh D, Horsley A. Feasibility and challenges of using multiple breath washout in COPD. Int J Chron Obstruct Pulmon Dis. 2018;13:2113-9.
5. Alton E, Armstrong DK, Ashby D, Bayfield KJ, Bilton D, Bloomfield EV, et al. Repeated nebulisation of non-viral CFTR gene therapy in patients with cystic fibrosis: a randomised, double-blind, placebo-controlled, phase 2b trial. Lancet Respir Med. 2015;3(9):684-91.
6. Robinson PD, Latzin P, Verbanck S, Hall GL, Horsley A, Gappa M, et al. Consensus statement for inert gas washout measurement using multiple- and single- breath tests. Eur Respir J. 2013;41(3):507-22.
7. Berlin KS, Parra GR, Williams NA. An introduction to latent variable mixture modeling (part 2): longitudinal latent class growth analysis and growth mixture models. J Pediatr Psychol. 2014;39(2):188-203.
8. Wickrama KA, Conger RD, Lorenz FO, Jung T. Family antecedents and consequences of trajectories of depressive symptoms from adolescence to young adulthood: a life course investigation. J Health Soc Behav. 2008;49(4):468-83.
9. Muthen B, Muthen LK. Integrating person-centered and variable-centered analyses: growth mixture modeling with latent trajectory classes. Alcohol Clin Exp Res. 2000;24(6):882-91.
10. Lee TK, Wickrama KAS, O'Neal CW, Lorenz FO. Social stratification of general psychopathology trajectories and young adult social outcomes: A second-order growth mixture analysis over the early life course. J Affect Disord. 2017;208:375-83.
11. Peugh J, Fan X. How Well Does Growth Mixture Modeling Identify Heterogeneous Growth Trajectories? A Simulation Study Examining GMM's Performance Characteristics. Structural Equation Modeling: A Multidisciplinary Journal. 2012;19(2):204-26.
12. Andruff H, Carraro N, Thompson A, Gaudreau P, Louvet B. Latent Class Growth Modelling: A Tutorial. Tutorials in Quantitative Methods for Psychology. 2009;5(1):11-24.
13. Clark S, Muthen B. Relating Latent Class Analysis Results to Variables not Included in the Analysis2009. Available from: https://www.statmodel.com/download/relatinglca.pdf.
14. van de Schoot R, Sijbrandij M, Winter SD, Depaoli S, Vermunt JK. The GRoLTS-Checklist: Guidelines for Reporting on Latent Trajectory Studies. Structural Equation Modeling: A Multidisciplinary Journal. 2017;24(3):451-67.
